# Role of Lipid Transfer Proteins (LTPs) in the Viral Life Cycle

**DOI:** 10.3389/fmicb.2021.673509

**Published:** 2021-06-23

**Authors:** Kiran Avula, Bharati Singh, Preethy V. Kumar, Gulam H. Syed

**Affiliations:** ^1^Virus-Host Interaction Lab, Institute of Life Sciences, Bhubaneshwar, India; ^2^Regional Centre for Biotechnology, Faridabad, India; ^3^School of Biotechnology, Kalinga Institute of Industrial Technology, Bhubaneshwar, India

**Keywords:** lipid transfer proteins, virus, replication, HCV, membrane contact sites, OSBP, CERT, NPC1

## Abstract

Viruses are obligate parasites that depend on the host cell machinery for their replication and dissemination. Cellular lipids play a central role in multiple stages of the viral life cycle such as entry, replication, morphogenesis, and egress. Most viruses reorganize the host cell membranes for the establishment of viral replication complex. These specialized structures allow the segregation of replicating viral RNA from ribosomes and protect it from host nucleases. They also facilitate localized enrichment of cellular components required for viral replication and assembly. The specific composition of the lipid membrane governs its ability to form negative or positive curvature and possess a rigid or flexible form, which is crucial for membrane rearrangement and establishment of viral replication complexes. In this review, we highlight how different viruses manipulate host lipid transfer proteins and harness their functions to enrich different membrane compartments with specific lipids in order to facilitate multiple aspects of the viral life cycle.

## Introduction

Biomolecules are the basic building block of life. Lipids are non-polar biomolecules that can be classified functionally into structural components of the cell (ex: phospholipids), energy storage molecules (ex: triglycerides), and signaling molecules (ex: steroid hormones). Lipid bilayers serve as limiting membranes of different cellular compartments that define their spatial limits and allows regulated exchanges between them. The specific lipid composition of the membranes dictates their functions, and intracellular lipid trafficking machinery helps in tailoring the unique composition of lipid membrane.

Viruses being obligate parasites are dependent on the host cell machinery for their replication and dissemination. Apart from being an integral part of the virus particle, cellular lipids play a central role in multiple stages of the viral life cycle. This includes: (i) binding and entry of the virus particle into the host cells by fusion of viral envelopes with cellular membranes, (ii) reorganization of host cell membranes for the establishment of viral replication compartments, and (iii) utilization of host lipid membranes and platforms for envelopment and egress of nascent virions (Zilversmit, 1983; Holthuis and Levine, 2005). Thus many viruses target host lipid synthesis and signaling to enrich the intracellular environment with lipids and promote membrane reorganization (Murillo et al., 2015). Viruses modulate host lipid signaling, trafficking, and synthesis to benefit the viral life cycle. In this review, we will discuss how the viruses manipulate the host lipid transfer proteins and harness their functions to facilitate multiple aspects of the viral life cycle and also highlight the importance of targeting host-lipid transfer proteins as an avenue for developing therapeutic strategies against viral infection.

## Lipid Transfer Proteins (LTPs)

Inter-and intra-cellular transportation of lipids is mediated by two distinct processes such as vesicular and non-vesicular mediated lipid transport. Cells usually prefer a non-vesicular mediated mode of lipid transfer for intracellular trafficking because unlike the vesicular process it is energy efficient and does not require cytoskeletal reorganization and complex signaling cascades (Das and Nozaki, 2018). Non-vesicular lipid transfer involves the exchange of monomeric lipids between the intracellular membranes and organelles. There are three mechanisms involved in non-vesicular lipid transport: monomeric lipid exchange, lateral diffusion and *trans* bilayer flip–flop. Lipid transfer proteins (LTPs) mediate monomeric lipid exchange by transferring a lipid to the acceptor membrane in exchange with a lipid of the acceptor membrane (Sleight, 1987; van Meer, 1989). Lateral diffusion occurs in the lateral plane of membrane bilayer usually occurring between membranes that are connected by membrane bridges (Sleight, 1987; Sprong et al., 2001). *Trans* bilayer flip–flop movement of lipids is spontaneous or mediated by proteins such as flippases and translocases (Perkins et al., 1997; Lev, 2006). *Trans* bilayer flip–flop movement does not majorly contribute to lipid transport between organelles but may indirectly influence inter-organelle lipid transport by other means (Chiapparino et al., 2016).

Lipid transfer proteins are a class of highly conserved proteins that facilitate the non-vesicular transfer of lipids from one organelle to another (Chiapparino et al., 2016; Wong et al., 2019). Around 125 genes in the human genome encode for the 10 different classes of LTP’s expressed in different human tissues (Chiapparino et al., 2016). The distinction of each family is based on the structural fold of their water-soluble and globular lipid transfer domain (LTD). Each family has LTDs comprising of either α-helices, β-sheets, or both. Lipids are accommodated inside a tunnel-shaped deep hydrophobic or amphiphilic cavity in the LTDs, which provides a hydrophobic environment to the non-polar group of the lipids (Wong et al., 2019). A flexible motif referred to as a “lid” or a “cap” often blocks the cavity entrance, and sterically disables the dissociation of the bound lipid ligand (Chiapparino et al., 2016). The ligand-binding specificity is created by a series of polar groups on the ligands such as the head groups of glycosphingolipids (GSLs) that engage in interim molecular hydrogen bonding to the LTD residues in the interior of the cavity (Malinina et al., 2004; Kudo et al., 2008). Targeting of LTPs to specific subcellular membranes often requires the integration of multiple low-affinity interactions. LTPs not only promote non-vesicular transfer of lipids between cellular organelles but also contribute in inter-organelle membrane tethering mechanisms by facilitating the formation of membrane contact sites (MCSs) (Hanada et al., 2003; D’Angelo et al., 2007, 2013; Mesmin et al., 2013). Through this function, the LTPs link the endoplasmic reticulum (ER), a major site of cellular lipid biogenesis to other organelles like Golgi-apparatus, Mitochondria, and Plasma membrane (Achleitner et al., 1999; Csordás et al., 2006; Elbaz and Schuldiner, 2011; Helle et al., 2013; Maeda et al., 2013). Both spontaneous and LTP mediated routes of lipid transfer are functional at MCS. The MCS not only facilitates the transport of lipids but also contributes to non-vesicular transfer and exchange of different metabolites/ions such as calcium ions between the cellular organelles (Levine and Munro, 2002; Wong et al., 2018; Peretti et al., 2020). MCS is composed of proteins involved in lipid biosynthesis and signaling and is stabilized through protein–protein or protein–lipid interactions between the opposing membranes (Giorgi et al., 2009; Lebiedzinska et al., 2009; Lev, 2010). The proximity between donor and acceptor membrane that occurs at MCSs reduces the diffusion distance of LTPs and accelerates the intermembrane lipid transfer (Peretti et al., 2020). For further details on LTPs, we suggest referring to many elaborate reviews published on this subject (Zilversmit, 1983; Holthuis and Levine, 2005; Lev, 2006; Elbaz and Schuldiner, 2011; Chiapparino et al., 2016; Hanada, 2018; Wong et al., 2019).

In this review, we briefly discuss about: (i) LTPs that have been implicated to play a vital role in the life cycle of animal viruses, (ii) Functional role of LTPs in the life cycle of these viruses and how viruses exploit them for their production and dissemination, and (iii) Targeting LTPs for therapeutic purpose and development of potential antiviral agents.

### Oxysterol-Binding Protein (OSBP) Related Proteins (ORPs)

**FIGURE 1:**
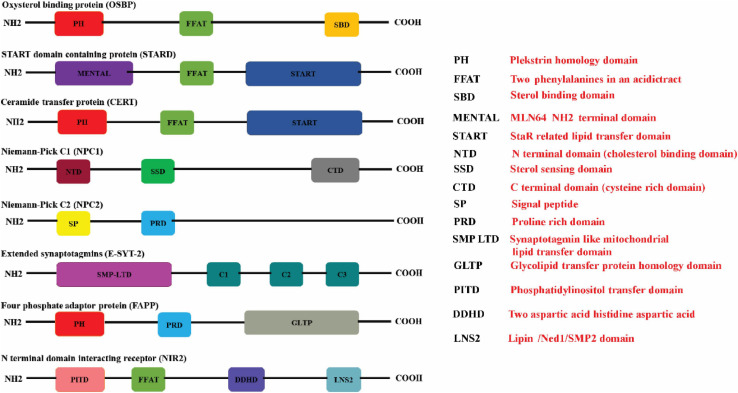
Schematic illustration of the protein domains in the respective lipid transfer proteins. The (red boxes) represent the Pleckstrin homology domain (PH domain) which binds to phosphatidylinositol-4-phosphate (PI4P) that is commonly present in OSBP, CERT, and FAPP. The FFAT motif which promotes interaction with ER-resident VAP proteins is represented in (green box) is present in OSBP, CERT, and STARD proteins. STEROL binding domain (dark yellow box), START domain (navy blue box), GLTP domain (gray box), and SMP domain (purple box) represents the characteristic lipid-binding domains specific for the individual LTPs. START proteins also have a multifunctional MENTAL domain (violet box) that binds to cholesterol. The E-Syts transfers glycerophospholipids through its mitochondrial-lipid-binding protein domain (SMP) (purple box). The E-Syts protein has a variable number of C2 domains (pine green boxes) which is responsible for Ca^2+^/phospholipid binding and protein–protein interactions. NPC1 has a cholesterol binding domain NTD (brown box), sterol sensing domain (SSD) (bottle green box) and a Cystine rich C terminal domain (dark gray box). Unlike NPC1, NPC2 consists of a signal peptide (butter yellow box) sensor and Proline rich domain (sapphire blue box). The proline rich domain is represented in (light blue box). The PITD of Nir2 is represented in (pink box) whereas LNS2 is represented as (teal colored box) (Created with BioRender.com).

Oxysterol-binding protein (OSBP) is a major representative member of the ORP family. All the ORPs contain a core OSBP-related domain (ORD). ORPs can bind sterols, however, some ORPs may preferentially bind to other lipids (Suchanek et al., 2007). Many ORPs also contain PH (Pleckstrin homology) domain that specifically binds to phosphatidylinositol-4-phosphate (PI4P), and FFAT [two phenylalanines (FF) in an acidic tract] motif that binds to the ER-resident VAMP-associated protein A (VAP-A) (Figure 1). Localization of ORPs is dynamic and oxysterol binding changes the subcellular localization of certain ORPs from the cytosol to the Golgi or ER (Ridgway et al., 1992). Although many ORPs can specifically bind to PI4P through the PH domain, some ORPs also bind to other phosphoinositides (Wong et al., 2019). OSBP can bind to both cholesterol and 25-hydroxycholesterol. Sterol transfer by OSBP is a cyclic process and involves the exchange of sterol from the ER with PI4P in opposing membranes (Ghai et al., 2017). The presence of PI4P on the donor membranes can stimulate OSBP activity (Ridgway et al., 1992). Loading/unloading of sterols into the LTD of OSBP modulates the opening and closing of the hydrophobic channel in LTD (Kwon et al., 2009).

### START (StAR-Related Lipid-Transfer) Domain Proteins

The steroidogenic acute regulatory protein (StAR)-related lipid transfer (START) domain is a protein domain spanning ∼210 residues that binds to lipids such as sterols (Ponting and Aravind, 1999). Fifteen mammalian proteins (STARD 1-15) have been identified to contain START domains (Alpy and Tomasetto, 2005) (Figure 1). The STARD1/D3 subfamily facilitates cholesterol transfer, STARD4/D5/D6 subfamily can bind to both cholesterol and oxysterol, STARD2/D7/D10/D11 subfamily facilitates the transfer of phospholipids and sphingolipids. The lipid ligands for other STARD family subgroups are currently unknown (Alpy and Tomasetto, 2005). STARD1 (also known as StAR) is a mitochondrial cholesterol carrier and facilitates cholesterol transfers from liposomes to mitochondria (Kallen et al., 1998). STARD3 (also known as metastatic lymph node 64, MLN64) plays a central role in the redistribution of cholesterol between the ER and endosomes (Soccio and Breslow, 2003). STARD3 anchored to the endosomal membrane interacts via its FFAT motif with the ER-resident VAPs (A and B) and MOSPD2 (motile sperm domain-containing 2) a VAP homolog protein to create the ER/endosomal contact sites. STARD3 captures cholesterol via its MENTAL domain (Figure 1) in the late endosome membranes, which are subsequently extracted by its cytoplasmic START domain and transferred to the receptor membrane in the cytosol (Alpy and Tomasetto, 2005). STARD 11 is commonly known as ceramide transfer protein (CERT), is involved in the transfer of ceramide, and N-acylated form of sphingosine, which serves as a key intermediate in the synthesis of sphingomyelin (SM), the plasma membrane sphingolipid, and various species of GSLs (Chiapparino et al., 2016). Sphingolipids are major components of lipid rafts and are required for the synthesis of signaling molecules such as sphingosine and sphingosine-1-phosphate (SIP) that play a vital role in cell signaling and physiology (Yamaji and Hanada, 2015). Ceramide synthesis takes place in the ER and transported to Golgi for further processing and subsequently distributed to various organelles. CERT is composed of three domains, N-terminal PH domain, C-terminal START domain, and a MR (middle region) domain (Fukasawa et al., 1999; Hanada et al., 2003; Yamaji and Hanada, 2014) (Figure 1). PH domain preferentially binds to PI4Pand plays a vital role in the localization of CERT to various membrane compartments (De Matteis et al., 2005). START domain of CERT specifically extracts ceramide and facilitates the transfer of ceramide between juxtaposing membranes (Hanada et al., 2003; Kumagai et al., 2005). Apart from these two domains, CERT also contains FFAT motif that interacts with the cytosolic region of the ER-resident VAP-A and VAP-B (Murphy and Levine, 2016) (Figure 1).

### Niemann-Pick Type C1 and C2 (NPC1 and NPC2) Proteins

Niemann Pick C (NPC) disease is an autosomal recessive disorder that exhibits impaired neuronal and visceral cell trafficking of cholesterol and other lipids, including glycolipids. Due to defect in the transport of cholesterol from the endosomal/lysosomal compartments the mutant cells exhibit accumulation of lipids in the late endosomes and lysosomes. NPC1 and NPC2 co-ordinate to export cholesterol from endosomal/lysosomal compartments. NPC1 is a membrane glycoprotein that resides primarily in the late endosomes and transiently localizes to lysosomes and TGN (Higgins et al., 1999; Neufeld et al., 1999; Patel et al., 1999). NPC1 contains 13 transmembrane domains, 3 large luminal hydrophilic loops, and a cytoplasmic tail (Kobayashi et al., 1999). Transmembrane domains 3–7 comprise the Sterol-Sensing Domain (SSD) of NPC1 (Long et al., 2020) (Figure 1). Along with SSD, NPC1 also has an MD-2-related lipid-recognition (ML) domain (Munkacsi et al., 2007). NPC2 is a soluble protein that binds to unesterified cholesterol and ML domain of NPC1 and transfers cholesterol to the N-terminal domain (NTD) of NPC1 (Li et al., 2016) (Figure 1). This transfer is facilitated by the formation of a channel between NTD of NPC1 that allows cholesterol to slide from NPC2 to NPC1 in a process known as a “hydrophobic handoff” (Kwon et al., 2009). NPC2 can also transfer cholesterol to phospholipid membranes very rapidly through a collision mechanism, which involves direct interaction with the acceptor membrane. NPC2 mediated transfer of cholesterol to membranes is very rapid in an acidic environment and enhanced by the presence of the unique lysosomal/late endosomal phospholipid lyso-bisphosphatidic acid (LBPA) (Ko et al., 2003; Cheruku et al., 2006). The complete details of how NPC1 and NPC2 mediate cholesterol transport from late endosomal/lysosomal compartments are still not clear.

### Extended Synaptotagmins (E-Syts Family)

The extended synaptotagmins (E-Syts) is a family of ER-resident LTPs having an N-terminal membrane anchoring domain and cytosolic exposed C2 domains that transfer glycerophospholipids between ER and plasma membrane (El Kasmi et al., 2018). The N- terminal region also contains the synaptotagmin-like mitochondrial and lipid-binding (SMP) domain) which is followed by a variable number of C2 domains in different members of the family. Mammals express three E-Syts (E-Syt1, E-Syt2, and E-Syt3). E-Syt 1 has five C2 domains and E-Syt2 and E-Syt3 have three C2 domains (Min et al., 2007) (Figure 1). The C2 domain is responsible for Ca^2+^/phospholipid binding and protein–protein interactions. E-Syts activities are controlled by Ca^2+^ and facilitates Ca^2+^dependent regulation of lipid transport and intracellular membrane transport (Yu et al., 2016). An increase in the intracellular Ca^2+^ mediates the enrichment of E-Syt1 from ER to ER–PM junctions (Pérez-Lara and Jahn, 2015). E-Syt1 hydrophobic moieties in the SMP domain form a hydrophobic channel leading to the bridge formation between opposing membranes and resultant transfer of glycerophospholipids in tunnel mode. Sometimes E-Syt 1 also transfers lipids by shuttle mode, which involves the extraction of lipids from ER and delivering it to the PM (Schauder et al., 2014). Currently, there is a lack of complete details on the exact mechanism involved in lipid transfer mediated by E-Syt proteins between the contact sites.

### Four-Phosphate Adaptor Protein 2 (FAPP2)

The four-phosphate adaptor protein 2 (FAPP2) is a member of glycolipid-transfer domain family (GLTD) (Hanada, 2018). FAPP2 localizes to the *trans*-Golgi network and facilitates the transportation of proteins from the Golgi complex to the cell surface. It is also involved in GSL metabolism at the Golgi complex (D’Angelo et al., 2012). FAPP2 has a PH domain with an additional globular domain at its carboxy terminus (Figure 1) (Godi et al., 2004). PH domain of FAPP2 bind selectively to PI4P and targets the protein to *trans*-Golgi network whereas the globular domain binds to glucosylceramide (GlcCer) primarily synthesized on the cytosolic leaflet of *cis*-Golgi. FAPP2 facilitates transfer of GlcCer from the *cis*-Golgi to the *trans*-Golgi regions or from the *cis*-Golgi to the ER (Rao et al., 2004).

### N-Terminal Domain-Interacting Receptor (Nir)

N-terminal domain-interacting receptor (Nir) is a member of phosphoinositide transfer protein (PITP) (Peretti et al., 2020). There are three isoforms of Nir encoded by the human genome, Nir1, Nir2, Nir3. Nir1 lacks an LTD in the N-terminal region which is present in Nir2 and Nir3. The structural prototype of Nir2/Nir3 LTD is a PI-transfer protein (PITP)-α isoform (PITPα) which belongs to PI-transfer domain family (Hanada, 2018). Nir2 has a canonical FFAT motif that enables its localization to ER (Figure 1). Nir2 has been shown to exchange PI (phosphatidylinositol) and PC (phosphatidylcholine) between the ER and Golgi apparatus and also PC and PA (phosphatidic acid) between the ER and PM, thus exerting dual function, i.e., PI/PC exchange at ER-Golgi MCSs and PI/PA exchange at ER-PM MCSs (Hanada, 2018). PI transfer by Nir2 is required for the maintenance of PI4,5P2 pools at the PM and the transfer of PA from PM to ER is tightly coupled to PI4,5P2 hydrolysis at PM and PI transfer to ER (Kim et al., 2015).

## Lipid Transfer Proteins in the Viral Life Cycle

Viruses promote membrane rearrangement to establish highly specialized replication compartments. These structures allow spatial segregation of replicating viral RNA from ribosomes and protect from host nucleases and pattern recognition receptors. In addition, by reducing the overall diffusion space these structures promote the enrichment of components required for viral replication and provide a conducive environment for virion assembly. Specific lipid composition of the membranes is required to facilitate the formation of negative or positive curvature and attributes membrane rigidity or flexibility to aid in membrane rearrangement. For instance, cholesterol a lipid of intrinsic negative curvature due to its small head group, and the large hydrophobic tail is enriched in membranes of the vesicles. Many viruses harness the ability of LTPs to promote the transfer of lipids from one membrane compartment to the other to enrich specific lipids that induce curvature and flexibility in membranes that make up the viral replication compartments.

### Hepatitis C Virus (HCV)

Hepatitis C virus (HCV) is a positive-sense ssRNA virus of the family *Flaviviridae*, genus *Hepacivirus*. About 71 million people worldwide are affected by HCV as per the World Health Organization (Shepard et al., 2005). HCV associated chronic hepatitis leads to hepatic steatosis that slowly progresses into liver fibrosis, cirrhosis, and hepatocellular carcinoma (HCC) (Negro and Sanyal, 2009). It has been reported that 5–25% of people with chronic hepatitis C develop cirrhosis within 10–20 years. Patients with cirrhosis have a 1–4% risk of developing HCC and 3–6% risk of hepatic decompensation (Thomas, 2013).

Hepatitis C virus replication factory comprises of a highly convoluted membranous structure known as the ‘membranous web’ derived from the ER membranes. The membranous web is majorly composed of double-membrane vesicles (DMVs) and multi-membrane vesicles (MMVs), which serve as a platform for HCV genome replication. HCV exploits several host factors to promote the enrichment of cholesterol in the replication compartment or membranous web HCV triggers fatty liver by upregulating lipogenesis and downregulating lipid secretion and β-oxidation (Syed et al., 2010). HCV non-structural protein 5A (NS5A) interacts with the ER-localized phosphatidylinositol-4 kinase III alpha (PI4KIIIα) and stimulates its kinase activity (Berger et al., 2009; Reiss et al., 2011). Activation of PI4KIIIα results in enhance levels of PI4P in the ER membranes and knockdown of PI4KIIIα leads to a defect in HCV replication and membranous web morphology (Reiss et al., 2011). PI4KIIIα inhibitor PIK93 reduced HCV-NS5A mediated induction of PI4P and affected the subcellular distribution of PI4P in HCV-infected cells (Reiss et al., 2011). PI4P recruits OSBP to the viral replication complex membranes. OSBP binds to PI4P as well as to VAP anchored at the ER membrane and catalyzes the exchange of cholesterol and PI4P between the ER and HCV replication organelles. OSBP is required for HCV replication and membranous web integrity and OSBP recruitment to the membranous web is PI4KIIIα dependent (Wang and Tai, 2019). HCV NS5A also interacts with the OSBP near the Golgi compartment and this interaction is shown to be essential for HCV egress (Amako et al., 2009). The phosphatidylinositol transfer protein Nir2 is also shown to support HCV replication. Nir2 facilitates the replenishment of PI4P pools at HCV membranous webs to maintain steady-state levels of PI4P and thus in co-ordination with VAP and OSBP enables the PI4P flow between the ER and membranous web to drive HCV replication (Wang and Tai, 2019). ORP4 (OSBP2) is a closely related paralog of OSBP and is found to negatively regulate HCV replication. ORP4S (a naturally occurring variant of ORP4 lacking the PH domain which is required to bind to PI4P) expression also results in inhibition of HCV pointing to an important role of the PH domain in HCV replication. ORP4 is also shown to interact with HCV NS5B (RNA dependent RNA polymerase) and inhibit HCV replication (Park et al., 2009).

**FIGURE 2:**
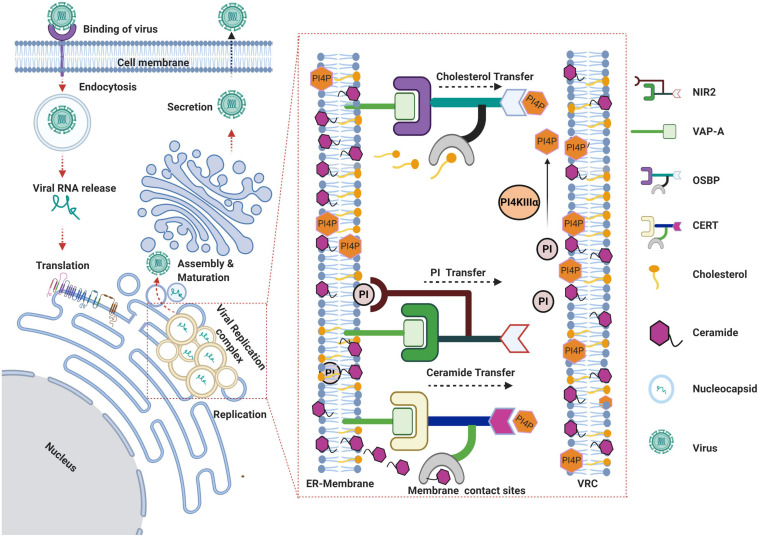
Schema representing the role of lipid transfer proteins in HCV life cycle. HCV virus gains entry via receptor-mediated endocytosis and subsequent uncoating of the viral envelope in the endocytic vesicles results in the release of viral genome into the cytoplasm. The viral RNA gets translated on the rough ER leading to the biosynthesis of viral polyprotein that is subsequently processed by host and viral proteases to yield structural and non-structural viral proteins. Viral proteins along with other host factors promote the rearrangement of ER-derived membranes to establish the viral replication complex. Recruitment and activation of PI4KIIIα at viral replication sites leads to enhanced levels of phosphatidylinositol-4-phosphate (PI4P) at replication sites resulting in recruitment of the lipid transfer proteins OSBP and CERT to the membranes. OSBP and CERT bind to PI4P via their Pleckstrin homology (PH) domains and to the ER anchored vesicle-associated membrane-associated protein (VAP) by FFAT motif. Both OSBP and CERT bind to their respective lipid ligands (i.e., cholesterol or ceramide) through their lipid binding domains and catalyze the transfer of cholesterol and ceramide respectively, in exchange for PI4P between the ER and virus replication complex. Nir2 facilitates replenishment of PI4P at HCV replication sites by coordinating the transfer of PI from ER membranes, which can be further converted to PI4P by PI4KIIIα. The assembly of viral genomic RNA into nucleocapsid takes place near the replication complexes followed by subsequent morphogenesis into virus particle and secretion through the Golgi secretory pathway (Created with BioRender.com).

Sphingolipids have been implicated to play vital role in HCV life cycle (Hanada et al., 2003; Aizaki et al., 2008; Amako et al., 2011). Some studies have shown that inhibition of sphingomyelin biosynthesis, either by small molecule inhibitor such as NA255 or by knocking out (KO) of CERT, suppressed HCV replication in a genotype-independent manner (Sakamoto et al., 2005; Gewaid et al., 2020) whereas other group has reported that the sphingolipid biosynthetic pathway inhibitor, HPA-12 blocks HCV virions production but not genome replication (Aizaki et al., 2008). FAPP2, a GLTP-like CERT is also implicated to play a role in HCV replication. FAPP2 is shown to localize to the HCV replication sites and promotes the transfer of lactosylceramide to the membranous web. Both PH and GLTP domains of FAPP2 are crucial for its role in HCV replication and FAPP2 depletion disrupts the HCV replication complex (Khan et al., 2014). Protein kinase D (PKD) regulates vesicular trafficking from *trans*-Golgi to the plasma membrane by mediating the phosphorylation-dependent inhibition of OSBP and CERT. PKD depletion or chemical inhibition enhanced HCV secretion suggesting that the transfer of lipids from ER to Golgi mediated by OSBP and CERT is required for maintenance of Golgi function and HCV egress (Amako et al., 2011). Subsequent studies highlighted the role of several other LTPs as host factors for HCV replication. Proteins such as STARD3, OSBP-related protein 1A, and -B (OSBPL1A and -B), and NPC1 have been shown to play a crucial role in HCV replication (Stoeck et al., 2018). Inhibition of NPC1 causes cholesterol entrapment in the lysosomal vesicles with a concomitant decrease in HCV replication compartment leading to the loss of membranous web integrity. This suggests that NPC1-mediated endosomal/lysosomal cholesterol transport is required for subsequent enrichment of cholesterol in the HCV membranous web (Stoeck et al., 2018). One study demonstrated that the Niemann-Pick C1-like 1 (NPC1L1) cholesterol uptake receptor is a host factor required for HCV entry. NPC1LI is commonly found in the bile canaliculus and localized on the apical membrane of hepatocytes. Chemical inhibition or silencing of NPC1L1 leads to inhibition of HCV infection *in vitro*. Most likely, the inhibition of NPC1LI results in the depletion of plasma membrane cholesterol which leads to defect in HCV uptake due to inhibition of HCV virion-cell membrane fusion (Sainz et al., 2012). Overall, these studies highlight the role of non-vesicular cholesterol transport at the ER and the late endosome/lysosome compartments plays a vital role in the maintenance of HCV membranous web integrity and optimal replication. An illustration depicting the role of some LTPs in HCV life cycle is shown in Figure 2.

### Dengue Virus (DENV)

Dengue virus (DENV) is a positive-sense ssRNA arbovirus of the family *Flaviviridae*, and genus *Flavivirus*. It has been reported that around 40% of the world’s population live in areas prone to DENV (Simmons et al., 2012). Annually almost 400 million people get infected with DENV (Centers for Disease Control and Prevention, 2019). Infection with any of the four DENV serotypes (DENV-1, DENV-2, DENV-3, and DENV-4) may lead to asymptomatic infection or result in a symptomatic infection that varies from mild febrile illness to severe Dengue fever associated with vascular fragility (dengue hemorrhagic fever) and hypovolemic shock (dengue shock syndrome) (Harris et al., 2000). Like many other viruses, DENV modulates the host cell metabolism and signaling pathways to benefit its replication and dissemination (Chatel-Chaix and Bartenschlager, 2014; You et al., 2015). Dengue genome replication occurs in viral replication complexes (VRCs) consisting of membrane packets, DMVs, tubular structures, and convoluted membranes (CM), derived by the reorganization of the host intracellular membranes (Welsch et al., 2009; Junjhon et al., 2014). DENV infection promotes the activity of 3-hydroxy-3-methyl-glutaryl-CoA reductase (HMGCR) and fatty acid synthase (FASN) enzymes resulting in enhanced *de novo* synthesis of fatty acids and cholesterol, which may be required for the formation of VRCs (Heaton et al., 2010; Soto-Acosta et al., 2017). Earlier studies have suggested that PI4P enrichment at replication sites is not required and PI4KIIIα, PI4KIIIβ, and OSBP activity is dispensable for Dengue replication (Negro and Sanyal, 2009; Thomas, 2013). PI4KIIIα inhibitor AL-9 and OSBP inhibitor OSW-1 had only minor effect on DENV replication at concentrations that strongly inhibit HCV replication (Wang and Tai, 2019). High-throughput screening of National Institutes of Health (NIH) Clinical Collection using an unbiased DENV replicon-based system to identify DENV inhibitors led to the identification of itraconazole (ITZ), a broad range inhibitor of human fungal infections (Cleef et al., 2013). Treatment with ITZ and its derivative posaconazole inhibited DENV replication. OSBP knockdown increased the sensitivity of DENV to posaconazole suggesting that inhibitory activity of antifungal posaconazole against DENV replication is mediated by OSBP, establishing OSBP as a host factor for DENV replication (Meutiawati et al., 2018). In contrast to previous studies, another group suggested that knockdown of OSBP, ORP2, and ORP11 or treatment with OSBP antagonist OSW1 inhibited DENV replication suggesting that OSBP and its related proteins 2 and 11 are required for DENV replication (Meutiawati et al., 2018). Unlike HCV, OSBP does not localize with DENV replication complexes suggesting that OSBP is not directly involved in DENV replication but supports replication by altering the intracellular lipid homeostasis (Meutiawati et al., 2018). To date, the functions of ORP2 and ORP11 are not fully resolved. It has been proposed that ORP11 lacks a known ER targeting signal and localizes on Golgi membranes and late endosomes to mediate non-vesicular lipid trafficking between these compartments (Zhou et al., 2010). ORP2, unlike OSBP and ORP11, does not possess a PH domain. Yet, ORP2 might regulate cellular sterol homeostasis and has been shown to have a role in intracellular cholesterol trafficking, endocytosis, and neutral lipid metabolism (Laitinen et al., 2002; Hynynen et al., 2009). The precise functions of these ORPs in the DENV replication cycle remain to be studied in detail. Interestingly, empirical evidence suggests that African descent is protective against dengue hemorrhagic phenotype in the Cuban population and two candidate genes, OSBPL10 and RXRA, have been implicated to confer protection (Sierra et al., 2017). Knockdown of OSBPL10 gene expression leads to a significant reduction in DENV2 replication. Although it is still not clear how OSBPL10 contributes to DENV replication, it is important to note that proteins involved in lipid metabolism play a crucial role in the DENV life cycle.

### Ebola Virus (EBOV) and Marburg Virus (MARV)

*Ebola virus* (EBOV), *Marburg virus* (MARV) belong to the *Filoviridae* family. These are filamentous, enveloped and negative-sense ssRNA viruses. Infection with EBOV causes Ebola virus disease (EVD) a fatal illness associated with coagulation disorders, hemorrhagic fever, lymphopenia, leading to septic shock and multi organ failure in affected humans and primates. MARV infection also leads to similar clinical sequelae as that of EBOV. Infected individuals experience severe watery diarrhea, abdominal pain, vomiting, and lethargy which progresses into severe hemorrhagic manifestation and shock. The average fatality rate of EVD is around 50% and about 90% in MARV infection (Schnittler and Feldmann, 2003; Maruyama et al., 2014). Filovirus genome expresses seven structural proteins, including a glycoprotein (GP), nucleoprotein (NP), viral proteins (VP) 24, VP30, VP35, VP40, and an RNA-dependent RNA polymerase. The EBOV-GP controls two critical aspects of viral entry: receptor binding and membrane fusion. Studies using global gene disruption in human cells led to the identification of the role of NPC1 in Filovirus entry (Carette et al., 2011). Cells defective for the homotypic fusion and protein sorting (HOPS) complex or NPC1 function, including primary fibroblasts derived from human NPC1 disease patients, are resistant to infection by *Ebola* and *Marburg* viruses (Carette et al., 2011). HOPS complex regulates the late endosomes maturation by mediating their fusion with lysosomes (Nickerson et al., 2009). Defect in HOPS complex may perturb the endo-lysosomal trafficking which may negatively impact Filovirus entry (Carette et al., 2011). NPC1-null cells are resistant to EBOV infection and the expression of human NPC1 reverses the resistance (Carette et al., 2011). Upon cleavage by cathepsin in late endosomes the receptor-binding domain (RBD) of the GP gets exposed thereby facilitating its interaction with its intracellular receptor NPC1 in the late endosomal/lysosomal compartments leading to membrane fusion and release of the viral genome. Computational analysis has shown that NPC1 utilizes two protruding loops to engage a hydrophobic cavity on the head of cleaved GP with exposed RBD resulting in a conformational change in the GP internal fusion loop and membrane fusion (Wang et al., 2016). Overall these studies suggest that filovirus requires the NPC1 to promote membrane fusion and viral escape from the vesicular compartment protein, independent of NPC1 role in cholesterol transport function from the endosomal/lysosomal compartments.

### Enteroviruses

Enteroviruses are positive sense, ssRNA virus of family *Picornaviridae*, genus *Enterovirus*. Many viruses such as *Poliovirus, Coxsackievirus, Echovirus*, and human *Rhinovirus* belong to the *Enterovirus* category. Clinical manifestations of *Enterovirus* infection includes poliomyelitis, meningitis, hand-foot-and-mouth disease, rhinitis and other respiratory diseases (Tapparel et al., 2013). *Poliovirus* 2BC and 3A proteins play an important role in membrane rearrangements and require PI4KIIIβ as an essential host factor, which promotes accumulation of PI4P lipids on replication organelle (RO) and facilitates its formation (Hsu et al., 2010; Arita et al., 2013; Arita, 2014). 25-hydroxycholesterol (25HC) a high-affinity ligand of OSBP and its analog enviroxime perturb *Poliovirus* replication by inhibiting OSBP and transcriptional inhibition of the SREBP/SCAP pathway suggesting OSBP-mediated transfer of cholesterol to poliovirus replication organelles is crucial for efficient replication (Arita et al., 2013). Replication of human *Rhinoviruses* (the causative agent of the common cold) also requires OSBP-mediated transfer of cholesterol in exchange of PI4P at MCSs (Roulin et al., 2014). RNAi mediated knockdown of Sac1, an ER resident PI4P phosphatase also resulted in inhibition of *Rhinovirus* replication. PI4P dephosphorylation may reduce the competition for sterol binding on OSBP and thereby enhance rhinovirus replication by stimulating cholesterol transfer (Roulin et al., 2014). However another study showed that OSBP mutant lacking the PI4P binding PH domain fails to localize near the viral replication sites highlighting the importance of OSBP binding to PI4P (Strating et al., 2015). The PI4P-cholesterol flux also promotes the coupling of other lipids into *Rhinovirus* replication organelles, such as phosphatidylcholine (PC) through Phosphatidylinositol transfer protein β (PITPβ), which enhances the complexity of lipids build up in the membranes and facilitates the formation of replication organelles. Similar to *Poliovirus*, *Coxsackievirus* has also been shown to reorganize the secretory pathway to promote the formation of PI4P lipid-enriched microenvironment in a PI4KIIIβ-dependent manner (Hsu et al., 2010). The viral RNA-dependent RNA polymerase of *Coxsackievirus* specifically binds to PI4P and promotes PI4P mediated assembly of the replication complex. 25HC binds to OSBP with higher affinity than cholesterol and locks OSBP in an inactive state and inhibits replication of both *Rhinoviruses* and *Coxsackievirus* B3. Itraconazole was recently found to inhibit *Enterovirus* replication by binding to OSBP (Strating et al., 2015). In agreement, other OSBP antagonists like 25HC, OSW-1, and T-00127-HEV2, also showed inhibitory activity against *Enterovirus* replication (Arita et al., 2013; Roulin et al., 2014; Albulescu et al., 2015). Both itraconazole and posaconazole can bind to NPC1 and perturbs NPC1 mediated cholesterol transport from late endosomes/lysosomes, this can lead to inhibition of *Enterovirus* entry as well as viral replication due to the overall effect on cellular cholesterol homeostasis (Shim et al., 2016). *Enterovirus* protein 3A binds and exploits the Acyl-coenzyme A binding domain containing 3 (ACBD3) protein to recruit PI4KIIIβ to the viral ROs to produce PI4P and promote OSBP-mediated cholesterol flux (Hsu et al., 2010; Greninger et al., 2012; Rönnberg et al., 2012; Sasaki et al., 2012; Nchoutmboube et al., 2013; Téoulé et al., 2013). ACBD3 is a Golgi resident protein that recruits PI4KIIIβ to the Golgi and *trans*-Golgi network (TGN) and stimulates its enzymatic activity to produce PI4P (Sohda et al., 2001; Baumlova et al., 2014; Boura and Nencka, 2015). Using infection models of *Enteroviruses* A71 (EV-A71), D68 (EV-D68), *Rhinovirus*, and *Poliovirus* various groups have demonstrated that 3A protein binds with GOLD domain of ACBD3 and knockdown of either ACBD3 or PI4KIIIβ perturbs replication of these viruses signifying the role of PI4P and OSBP in enterovirus replication (Greninger et al., 2012; Rönnberg et al., 2012; Nchoutmboube et al., 2013; Téoulé et al., 2013; Lei et al., 2017; Lu et al., 2020). A recent study contradicted the above finding, and shown that *trans*-rescue of EV-A71 pseudo virus replication with PI4KIIIβ deletion mutants suggested that the ACBD3-binding site of PI4KIIIβ is not essential for EV-A71 or poliovirus replication (Arita, 2019).

### Aichi Virus (AiV)

Aichi virus (AiV) is a positive-sense single-stranded RNA virus of the *Picornaviridae* family and genus *Kobuvirus* (Rivadulla and Romalde, 2020). It is transmitted through the consumption of contaminated food and water and affects children below 5 years of age causing acute gastroenteritis (Rivadulla and Romalde, 2020). AiV hijacks intracellular cholesterol transport by exploiting OSBP to enrich cholesterol at viral replication sites in exchange for PI4P (Sasaki et al., 2012). Similar to enteroviruses, AiV interacts with ACBD3 to recruit PI4KIIIβ to the replication sites and enhance PI4P synthesis (Ishikawa-Sasaki et al., 2014). OSBP, VAP-A/B, and Sac1 co-localize to the replication organelles and serve as essential host factors for AiV replication (Ishikawa-Sasaki et al., 2018). AiV proteins 2B, 2BC, 2C, 3A, and 3AB bind with ACBD3 and PI4KIIIβ at replication sites to enhance PI4P levels which in turn leads to OSBP mediated cholesterol enrichment required for the formation of replication organelles (Ishikawa-Sasaki et al., 2018). AiV replication is inhibited upon silencing OSBP and the ER-resident VAP-A/B due to defect in OSBP-mediated cholesterol transfer at replication organelle.

### African Swine Fever Virus (ASFV)

African swine fever virus (ASF), is a large dsDNA virus of the *Asfarviridae* family and causes hemorrhagic fever in pigs with a high mortality rate. Initially, it was endemic in Africa but has slowly spread to Europe, China, and Southeast Asia causing huge socioeconomic losses (Karger et al., 2019). The viral replication takes place in the cytoplasm but is also reported to occur in the nucleus during the initial stages of infection (García-Beato et al., 1992; Rojo et al., 1999). ASFV remodels the intracellular membranes into viral replication factories (VF). ASFV promotes the redistribution of the intracellular vesicular system and redirects endosomal traffic to the early replication site resulting in the accumulation of endosomal membranes that provide membrane source and cholesterol supply required to establish the VF (Cuesta-Geijo et al., 2017). The cholesterol flux to the ASFV viral factories at the MCSs is mediated by a number of proteins such as the ACBD3, PI4KIIIβ, and the LTPs, OSBP and OSBP related proteins (ORP), which have high affinity to oxysterols and cholesterol (Galindo et al., 2019). ASFV utilizes ACBD3 to recruits PI4KIIIβ to VF promoting the accumulation of PI4P that triggers the redistribution of OSBP to the periphery of VFs and promotes cholesterol flux to the VFs. Itraconazole and 25-hydroxycholesterol (25-HC) has been shown to inhibit ASFV replication by blocking OSBP activity (Galindo et al., 2019).

### Adenoviruses (AdV)

Mammalian *Adenovirus* is a non-enveloped, dsDNA virus of the family *Adenoviridae*, genus *Mastadenovirus*. Adenoviruses commonly cause lower respiratory tract infection which is infrequent and sporadic in nature (Masci and Wormser, 2005). Virus infection is generally persistent and can be detected months after primary exposure (van der Veen and Lambriex, 1973; Horvath et al., 1986). *Adenovirus* infection in young children leads to gastrointestinal and respiratory symptoms or a combination of both. *Adenovirus* symptoms are mild and self-limiting but considerable morbidity and death have been observed in the pediatric population (Singh-Naz and Rodriguez, 1996; Mahr and Gooding, 1999; Walls et al., 2003). Human AdV is classified into six groups (HAdV-A to HAdV-F) based on their physical, biological, and chemical properties (Ghebremedhin, 2014; Lion, 2014). *Adenovirus* type 2 and type 5 (AdV2 and AdV5) of group C primarily target epithelial cell lining of the upper respiratory tract through binding to *Coxsackievirus* B adenovirus receptor (CAR) and αv integrin co-receptors (Meier and Greber, 2004). The viruses uncoat at plasma membrane thereby releasing the viral lytic protein VI that forms lesion on the plasma membrane leading to calcium influx and activation of canonical repair mechanism involving lysosome fusion with the plasma membrane. This leads to the release of acid sphingomyelinase (ASMase) into the extracellular space which in turn promotes localized degradation of sphingomyelin and generation of ceramide lipid in the outer plasma membrane leaflet thereby causing invagination and subsequent penetration and escape of viral capsid from endosome to the cytosol (Burckhardt et al., 2011; Luisoni et al., 2015). The adenovirus E3 transcript product RIDα is a small 13.7-kilodalton protein that attenuates EGF receptor (EGFR) signaling by promoting EGFR accumulation in pre-lysosomal compartments and subsequent degradation (Hoffman and Carlin, 1994). RIDα is also associated with attenuation of pro-inflammatory NFκB signaling induced by stress-activated EGF receptors (Zeng and Carlin, 2019). RIDα also modulates cholesterol homeostasis by regulating cholesterol transport from endosomes to the ER (Cianciola et al., 2013). RIDα rescues NPC1-deficient cells from accumulation of lysosomal storage organelles, by diverting excess free cholesterol to lipid droplets (Cianciola and Carlin, 2009). RIDα directly interacts with the sterol-binding protein ORP1L, a member of the OSBP-related proteins (ORPs) family and with ER-resident VAP proteins through its FFAT motif and supports the transport of LDL-derived cholesterol from endosomes to the ER, where it gets converted to cholesteryl esters and stored in lipid droplets. However, unlike NPC1 mediated cholesterol transport to ER and regulation of SREBP transcription factor, RIDα mediated cholesterol transport is sensitive to ACAT inhibition and correlates with induction of lipid droplets formation in adenovirus-infected cells (Cianciola and Carlin, 2009; Cianciola et al., 2013, 2017). Thus adenovirus RID-α restores the perturbed cholesterol balance during the early stages of acute infection and downregulates pro-inflammatory signaling pathway by innate immune toll-like receptor 4, which plays a major role in AdV pathogenesis.

### Herpes Simplex Virus Type 1 (HSV-1)

*Herpes Simplex* type 1 (HSV-1) is a linear dsDNA enveloped virus of the family *Herpesviridae*, subfamily *Alphaherpesvirinae*, genus *Simplex virus*. The pathogenesis of HSV-1 infection follows a cycle of infection which involves infection of epithelial cells followed by latency in neurons, and finally reactivation with symptoms such as primary and recurrent vesicular eruptions generally in the orolabial and genital mucosa (Pires de Mello et al., 2016). The herpes viruses undergo multiple distinct membrane fusion events during the multiples stages of life cycle such as entry and release of nascent virions. Herpes virus entry involves receptor binding and membrane fusion with plasma membrane resulting in the release of the nucleocapsid in the cytoplasm and subsequent delivery of the viral genome into the nucleus. The assembly of new nucleocapsid occurs in the nucleus, which are too large to get through the nuclear pores and bud into inner nuclear membrane resulting in transiently enveloped nascent virions (Padula et al., 2009; Wright et al., 2009). Subsequently, these virions fuse with the outer nuclear membrane releasing the naked nucleocapsid into the cytoplasm followed by their re-envelopment while fusion into Golgi compartment. While the virions bud out of the Golgi compartment they acquire a double-envelope, which is lost when the viruses fuse with plasma membrane leading to release of single enveloped mature virions (Siminoff and Menefee, 1966; Stackpole, 1969; Henaff et al., 2012). The membrane fusion functions are performed by multifunctional viral glycoproteins (Connolly et al., 2021). HSV-1 glycoprotein M (gM), a recognized modulator of virus-induced membrane fusion, interacts with the E-Syt1 host protein. E-Syt1 is a membrane fusion regulator that belongs to the extended synaptotagmins family (El Kasmi et al., 2018). E-Syt1 facilitates ER-PM tethering and is known to regulate many potential fusion steps of HSV-1 during the viral life cycle. Knocked down of E-Syt1 is associated with increased secretion of infectious virions and depletion of cell-associated viral particles suggesting that E-Syt1 negatively regulates virus release. In agreement, overexpression of E-Syt proteins E-Syt1 and E-Syt3 caused inhibition of virus release (El Kasmi et al., 2018). Transmission electron microscopy revealed that knockdown of E-Syt1 resulted in a modest and statistically significant decrease of the virus particles in the nucleus and cytoplasm with a concomitant increase at the cell surface. Currently, it is not clear if the glycerophospholipids transfer activity of E-Syt plays any role in the negative regulation of HSV release (El Kasmi et al., 2018).

### Chikungunya Virus (CHIKV)

*Chikungunya*, is a positive-sense ssRNA arbovirus of genus *Alphavirus* and family *Togaviridae* (Wong et al., 2021). As per WHO report the disease occurs mainly in Africa and Asia (WHO). Clinical manifestation of CHIKV infection is characterized by high fever, intense myalgia and arthralgia, nausea, fatigue, and rash (in 50% of the cases) (Wong et al., 2021). CHIKV is internalized by receptor mediated endocytosis (Lee et al., 2013). Presence of cholesterol in the plasma membrane has been shown to be essential for entry/fusion of a wide range of RNA viruses such as members of *Filovirus*, *Alphavirus*, and *Flavivirus* (Medigeshi et al., 2008; Rothwell et al., 2009; Herbert et al., 2015). Methyl-β cyclodextrine mediated depletion of cholesterol lead to inhibition of CHIKV entry and/or fusion (Bernard et al., 2010). In agreement, CHIKV infection of primary fibroblasts from patients with NPC disease, harboring mutations in the NPC-1 or NPC-2, lead to reduce levels of CHIKV infection and total viral output suggesting that cholesterol plays a crucial role in CHIKV life cycle. The infection of mutant fibroblasts with CHIKV-glycoproteins pseudo typed particles also demonstrated that both NPC-1 and NPC-2 deficiency affect viral entry/fusion events (Wichit et al., 2017)

## Antivirals Targeting LTPs

We have already discussed how viruses exploit the LTPs to facilitate different steps of their life cycle. Different viruses utilize the LTPs in a distinct manner to promote the establishment of viral replication complexes or facilitate viral entry and release. The LTPs not only facilitate the transfer of specific lipid but also recruit specific host/viral proteins to the viral replication factories. Targeting LTPs may provide potential avenues to develop specific and pan-viral inhibitors (as mentioned in Table 1). For instance, the antiviral effect of the interferon-inducible transmembrane protein 3 (IFITM3) on viral entry is mediated by the disruption of VAP-A and OSBP interaction resulting in deregulated cholesterol homeostasis and marked accumulation of cholesterol in multi vesicular bodies and late endosomes. The resultant defect in cholesterol trafficking results in defective fusion of viral membranes with endosomal compartments resulting in the blocking of virus release into the cytosol (Amini-Bavil-Olyaee et al., 2013). However, we need to pay attention that these strategies do not perturb cellular lipid homeostasis and lead to toxicity.

**TABLE 1:** Antivirals targeting lipid transfer protein.

**Virus**	**LTP**	**Target step**	**Inhibitor**	**References**
**HCV**	OSBP	Replication	Itraconazole Posaconazole OSW1	Sohda et al. (2001) Sohda et al. (2001) van Meer (1989)
**HCV**	GLTP	Replication	Bicyclol	Shim et al. (2016)
**HCV**	CERT	Secretion	HPA-12	Yasuda et al. (2001), Aizaki et al. (2008)
**HCV**	NPC1L1	Entry	Ezetimibe	Sainz et al. (2012)
**Enteroviruses**	OSBP	Replication	Itraconazole TTP-8307 OSW1	Sohda et al. (2001) Sun et al. (2015) Albulescu et al. (2017)
**DENV**	OSBP	Replication	Posaconazole OSW1	Sierra et al. (2017) Burgett et al. (2011)
**EBOV**	NPC1	Entry	U18666A 3.47 MBX2254 MBX2270	Wichit et al. (2017) Côté et al. (2011) Basu et al. (2015)
**CHIKV**	NPC1	Entry	Imipramine	Wichit et al. (2017)

**FIGURE 3:**
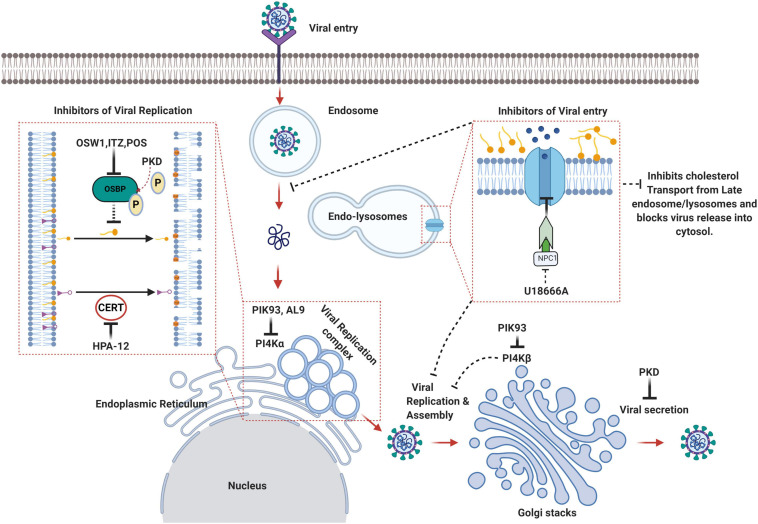
Schematic summary of the lipid transfer proteins as attractive therapeutic avenues against viruses. OSW1, ITZ (Itraconazole), POS (Posaconazole) binds to OSBP and inhibits its lipid shuttling activity at the ER-Golgi or ER-Viral replication membrane contact sites resulting in the disruption of viral replication sites. HPA-12 is a CERT inhibitor that inhibits ceramide trafficking from the ER to the site of sphingomyelin synthesis. PIK93 can inhibit both PI4KIIIα and PI4KIIIβ whereas AL9 inhibits PI4KIIIα thereby inhibiting PI4P production and recruitment of OSBP and CERT, which eventually affects non-vesicular lipid transport and viral replication sites integrity. U18666A binds to the luminal loop of NPC1 and blocks NPC1 mediated cholesterol flux from late endosomes/lysosomes thereby affecting viral entry and release into the cytosol (Created with BioRender.com).

Spontaneous interruption of HCV replication has been observed by treatment with bicyclol an active compound in traditional Chinese medicine that induces glycolipid transfer protein (GLTP) expression (Huang et al., 2019). HCV NS5A plays a key role in virus replication by activating the PI4KIIIα kinase and binding with OSBP and VAP in the ER (Albulescu et al., 2017). The binding site of GLTP to VAP-A overlaps with that of the NS5A binding site. VAP-A has a higher affinity to GLTP than NS5A, hence the induction of GLTP expression by bicyclol blocks the interaction between VAP-A and NS5A, causing a defect in the assembly of the HCV replication complex (Huang et al., 2019). CERT inhibitor, HPA-12, has also been found to suppress HCV secretion but not viral RNA replication due to the inhibition of ceramide trafficking from the ER to Golgi, the site of sphingomyelin synthesis (Yasuda et al., 2001; Aizaki et al., 2008). Itraconazole (ITZ) is an antifungal agent, which has been shown to have anti-cancer as well as antiviral properties. ITZ can bind to both OSBP and ORP4. ITZ inhibits the cholesterol and PI4P transfer function of OSBP. This leads to defect in virus-induced membrane alterations and establishment of viral replication complexes. ITZ exhibits broad-spectrum antiviral activity against many enteroviruses such as *Poliovirus, Coxsackievirus, Enterovirus-71 and Rhinovirus*. In agreement overexpression of OSBP reversed inhibitory effect of ITZ (Strating et al., 2015). TTP-8307, an another OSBP inhibitor similar to ITZ also exhibits anti-enteroviral potential (Albulescu et al., 2017). Posaconazole (POS) a derivative of ITZ has been effectively used to inhibit DENV replication by targeting OSBP. Knockdown of OSBP further sensitized the DENV-infected cells to the antiviral activity of POS and ITZ. DENV replication is also inhibited by OSW-1, a well-established ligand, and an inhibitor of OSBP (Meutiawati et al., 2018). OSW-1, an anti-cancer compound extracted from the bulbs of the plant *Ornithogalum saundersiae*, also has a high affinity for OSBP (Burgett et al., 2011). OSW-1 exhibited anti-viral activity against different species of enteroviruses by inhibiting OSBP function (Albulescu et al., 2017). U18666A is a cationic amphiphilic drug, which interacts with the luminal loop 2 of NPC1. NPC1 plays a major role in the entry of *Ebola* and *Marburg* viruses by interacting with the viral spike glycoprotein (Lu et al., 2015). The U18666A binding region on NPC1 overlaps with the viral glycoprotein binding site and hence blocks the interaction between the viral glycoprotein and NPC1 thereby inhibiting *Ebola* and *Marburg* virus entry (Lu et al., 2015). NPC1 inhibition by U18666A may perturb cholesterol transport into the cytosol from the endosomal/lysosomal compartments thereby leading to accumulation of cholesterol in late endosomes and lysosomes (Schloer et al., 2019). The OSBP inhibitors ITZ and POS have also been shown to interrupt NPC1 mediated cholesterol flux from the endosomal/lysosomal compartments (Shim et al., 2016). The resultant dysregulation in the intracellular cholesterol homeostasis may affect both the viral entry and replication events due to overall defects in: (i) membrane fusion required for viral entry and (ii) rearrangement of membranes for the establishment of the viral replication complexes. Imipramine, a class II cationic amphiphilic drug that mimics the molecular phenotype of NPC1 by blocking cholesterol exit from the late endosomes to the cell membrane is effective in inhibition of CHIKV replication in the primary human epidermal fibroblasts by interrupting intracellular cholesterol trafficking (Wichit et al., 2017). A schematic illustration of the therapeutic targeting of LTPs to inhibit the multiple stages in a viral life cycle is depicted in Figure 3.

## Concluding Remarks

There are still some lacunae in our understanding of how the LTPs function and how they co-ordinate with other LTPs and other modes of lipid transport to maintain intracellular lipid trafficking. Detailed studies of these aspects will provide clear insight into intracellular lipid trafficking and homeostasis. The establishment of specific replication structures is central to the life cycles of many RNA viruses and a clear understanding of the virus-host interactions that govern membrane rearrangement and assembly of these complex structures will enable the development of therapeutic strategies with pan-viral potential. Paradoxically many viruses exploit these common host factors to build structures that are very unique to individual viruses and drastically differ in shape and size. It is still an enigma how the viruses build such atypical structures and how the LTPs contribute in terms of recruiting other essential factors and lipids to the replication sites. Many studies suggest upregulation of autophagy during viral infection and the cross-talk between the LTPs and autophagy would be an interesting area to explore.

## Author Contributions

KA, BS, and PVK drafted the manuscript. KA, BS, and GHS improved the manuscript significantly by extensive discussion, addition and revision. GHS had edited the manuscript. All authors approved the submitted version.

## Conflict of Interest

The authors declare that the research was conducted in the absence of any commercial or financial relationships that could be construed as a potential conflict of interest.
